# A Rare Case of Anterior Stafne Bone Cavity in the Mandibular Region

**DOI:** 10.7759/cureus.97395

**Published:** 2025-11-20

**Authors:** Hideki Suito, Yuuri Oku, Koichi Kani, Keiko Aota, Naoki Maeda

**Affiliations:** 1 Department of Oral and Maxillofacial Radiology, Graduate School of Biomedical Sciences, Tokushima University, Tokushima, JPN; 2 Department of Oral Medicine, Graduate School of Biomedical Sciences, Tokushima University, Tokushima, JPN

**Keywords:** lingual mandibular bone defect, mandibular anterior region, multi-detector computed tomography, stafne bone cavity, static bone cavity

## Abstract

Stafne bone cavity (SBC) is a rare, asymptomatic mandibular bone defect that is usually detected incidentally on imaging. While the posterior variant is relatively common and typically occurs near the mandibular angle, the anterior form involving the incisor-premolar region is extremely rare and may mimic odontogenic or non-odontogenic cysts. An 81-year-old female was referred for evaluation of a swelling in the right maxilla. Panoramic radiography incidentally revealed a well-defined radiolucency in the left anterior-premolar mandibular region. Multi-detector CT demonstrated a lingual alveolar bone concavity measuring 20.9 × 8.7 × 11.2 mm with buccal cortical thinning, which was classified as Type Ⅱ according to Ariji’s criteria. Surgical exploration confirmed a lingual cortical depression without cystic or tumorous tissue, consistent with anterior SBC. No biopsy was performed, and the patient was managed conservatively with periodic follow-up. This case highlights the importance of considering anterior SBC in the differential diagnosis of radiolucent mandibular lesions and emphasizes the role of advanced imaging in establishing an accurate diagnosis and avoiding unnecessary invasive treatment.

## Introduction

Stafne bone cavity (SBC), also termed static bone cavity, latent bone cavity, or idiopathic bone cavity, is an asymptomatic and largely non-progressive bone defect incidentally discovered in the mandible during imaging studies. First reported by Edward Stafne in 1942 [1], SBCs are typically located in the mandible of the lingual side at the angle region posterior to the mandibular canal. The bone defect often contains abundant salivary gland tissue, though the presence of muscle tissue, lymphoid tissue, and blood vessels has also been documented [2]. Prevalence in the general population ranges from 0.1% to 0.5%, with a higher incidence reported in middle-aged males [3].

Posterior types are relatively easily diagnosed due to their typical location, but anterior types forming in the anterior or premolar regions are extremely rare among SBCs. Since their first description by Richard and Ziskind in 1957 [4], their incidence has been reported to be 0.009% [5]. Unlike the posterior type, the anterior type, due to its specific location, may be misinterpreted on panoramic radiographs as a dentigerous cyst, a non-dentigerous cyst, or even a benign tumor, presenting diagnostic challenges.

The advent of advanced imaging techniques such as CT (cone-beam CT) and MRI has enabled confirmation of the continuity between the SBC and adjacent salivary gland tissue, thereby avoiding unnecessary surgical intervention [6]. However, due to its rarity, reported cases remain limited, and further case reports are essential to deepen understanding of its clinical and imaging characteristics.

This report presents a rare case of SBC arising in the anterior mandible (incisor/premolar region), demonstrating its characteristic radiographic findings and the importance of differential diagnosis. It aims to contribute to the avoidance of unnecessary surgical procedures.

## Case presentation

In October 2024, an 81-year-old female patient was referred to our hospital’s Department of Oral Medicine from a local dental practice due to swelling of the gingiva around the right maxillary premolar and the right cheek. A panoramic radiograph taken for detailed examination revealed radiolucent lesions extending from the anterior mandible to the premolar region on the opposite side to the chief complaint. The patient reported no symptoms on the left side and had no history of trauma, swelling, or sensory abnormalities there. Extraoral and intraoral examination of this area showed no abnormalities, and the overlying mucosa appeared normal.

**Figure 1:**
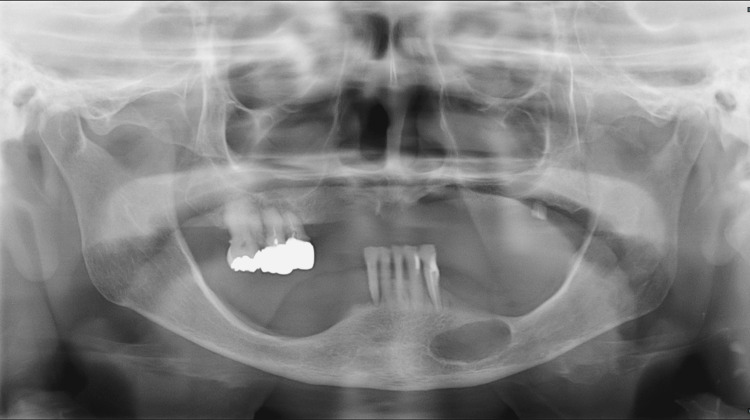
Panoramic X-ray on the initial visit. A clearly defined, internally homogeneous radiolucent area is observed from the mandibular region to the anterior area.

The mandibular retained teeth were 21┐┌123. The panoramic radiograph revealed a well-defined, single-chambered, elliptical radiolucent image extending from the left mandibular anterior region to the premolar region. The mesial portion of the lesion was located subapically to the root of the residual mandibular tooth, but it was spaced apart from the tooth apex, and the periodontal ligament space of the residual tooth was preserved (Figure 1).

Multidetector CT (MDCT) was performed using an Aquilion ONE/ViSION Edition (320 rows; Canon Medical Systems Co., Tochigi, Japan) with a tube voltage of 120 kV, tube current of 300 mA, field of view of 160 mm, rotation time of 0.5 s/rotation, and helical scanning. The reconstruction slice thickness was 0.5 mm, with a reconstruction interval of 0.5 mm.

**Figure 2:**
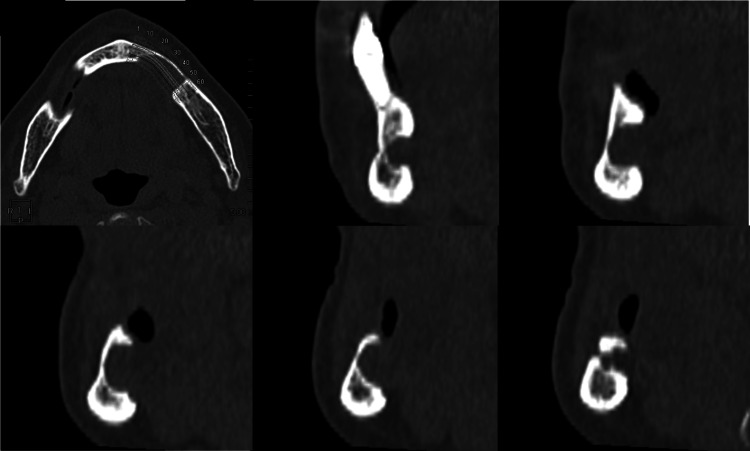
Multidetector CT image (hard tissue mode). A single-vesicular cavity is observed, possessing a cortical margin and opening toward the lingual side. The margin is relatively distinct, sharp, and regular.

**Figure 3:**
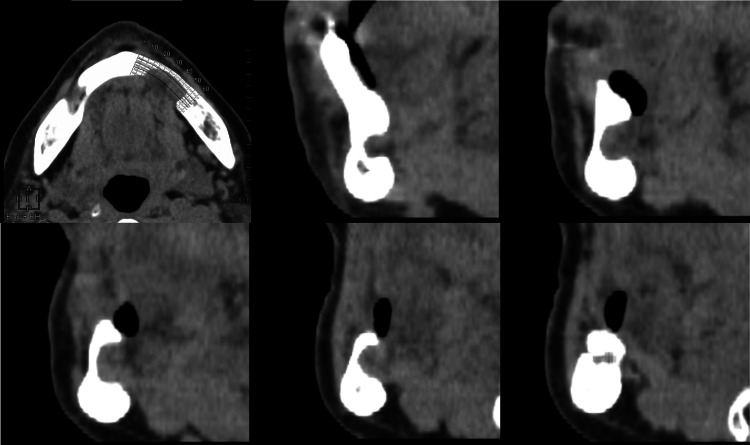
Multidetector CT image (soft-tissue mode). Continuity is observed between the internal tissue of the Stafne bone cavity (SBC) and the surrounding tissue. Tissue with differing X-ray absorption is present within the SBC.

**Figure 4:**
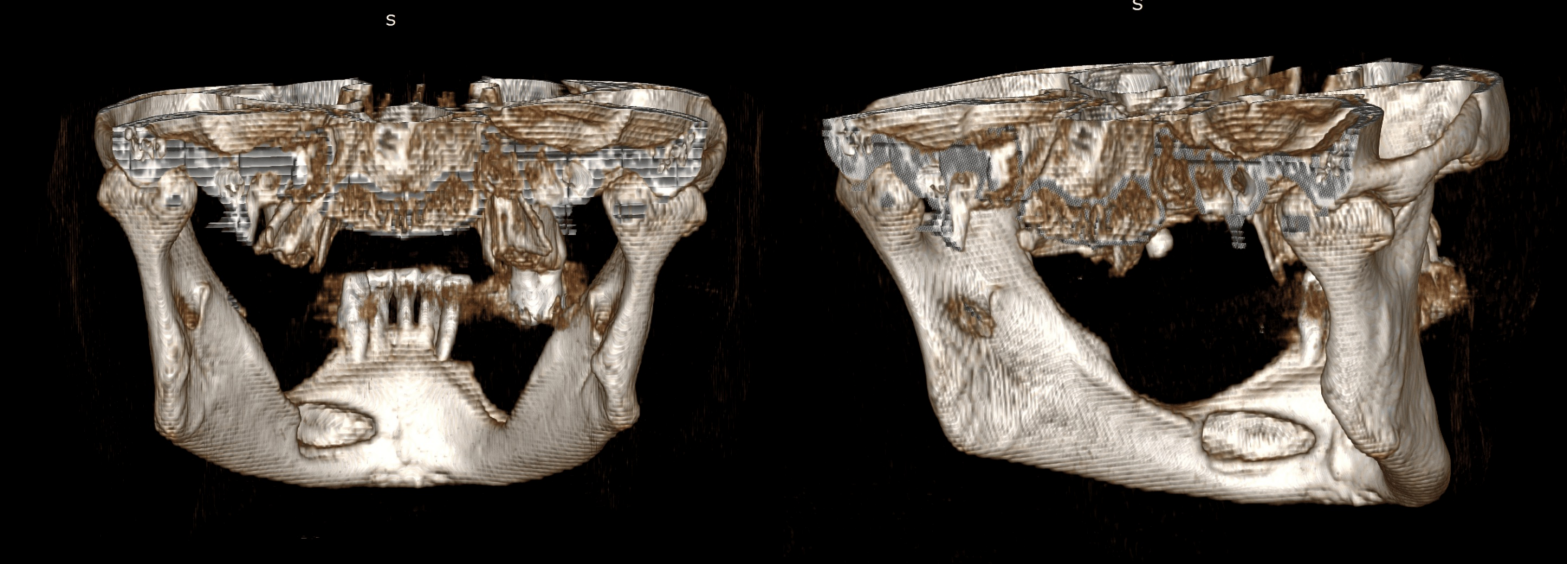
Three-dimensional model created from CT data. Left: Anterior view of the mandible. Right: Left anterior view of the mandible.

MDCT images taken in November 2024 (Figures 2-4) revealed a cortical bone depression measuring approximately 20.9 × 8.7 × 11.2 mm on the lingual aspect of the left mandible. The depression extended to the buccal cortical bone, with no thinning of the cortical bone or bulging of the buccal alveolar bone.

It was diagnosed as Type Ⅱ according to Ariji et al.’s classification (Table 1) [7]. The CT values within the depression ranged from -80 to 136 HU, showing a large standard deviation (SD), consistent with previous reports of anterior-type SBC [8]. The mandibular defect was classified as Type Ⅱ in the defect morphology classification and Type G in the defect contents classification, diagnosed using Ariji et al.’s classification (Table 1) [7]. The mandibular canal was displaced buccally, running adjacent to the SBC as if avoiding it, and opened buccally at the ┌5 equivalent region.

**Table 1:** The classification of Stafne bone cavities proposed by Ariji et al.

Type	Classification of concavities according to their outline and relationship to buccal cortical plate	Type	According to the content
Ⅰ	The bottom of the concavity did not reach the buccal cortical plate	F	The concavity is filled with only fat density
Ⅱ	The bottom of the concavity reached the buccal cortical plate, but there was no expansion or distortion of the plate	S	The concavity is filled with soft-tissue structure (lymph node, vessel, connective tissue, etc.)
Ⅲ	The bottom of the concavity reached the buccal cortical plate, and buccal expansion of the cortical plate	G	The concavity is filled with some glandular tissue (not exclusively the submandibular gland)

In April 2025, an incision was made at the alveolar crest for biopsy, and a mucoperiosteal flap was elevated and everted. This revealed that the mandible was concave from the lingual side. The subperiosteal area appeared normal (the cortical bone extended continuously from the alveolar crest to the depression surface), suggesting a state of compression leading to bone resorption. This was not the typical pathology of a mandibular cyst, where enlargement within the alveolar bone causes resorption of the lingual cortical bone. When the mucoperiosteal flap was detached and everted, no soft tissue was present within the bony recess, and a salivary gland-like soft-tissue bulge was observed on the mucoperiosteal flap. As no obvious cystic or tumor-like lesions were identified internally, a lingual defect was identified, confirming the diagnosis as an SBC. Consequently, to minimize patient invasiveness and the patient’s wish, a biopsy in content of the Stafne bone was not performed. The incision was closed, and the patient was placed under follow-up observation. It has been one year since then, with no swelling or infection at the surgical site, and the patient’s progress has been favorable. Regular follow-up examinations are planned going forward.

## Discussion

SBC is a mandibular bone defect commonly observed inferior to the mandibular canal in the posterior mandible of the lingual side. This lesion is typically asymptomatic and non-progressive, usually being discovered incidentally on panoramic radiography. The most clinically important consideration is differentiating SBC from other radiolucent lesions of the mandible and determining the necessity for surgery.

Particularly when exhibiting atypical morphology (e.g., lobulated, multiple, multicystic, or distinct internal components), diagnosis may not be possible with panoramic radiography alone. Differential diagnoses include traumatic bone cyst, simple bone cyst, periapical cyst, dentigerous cyst, odontogenic keratocyst, nonossifying fibroma, fibrous dysplasia, ameloblastoma, metastasis, giant cell tumor, vascular malformation, focal osteoporotic bone marrow defect, basal cell nevus syndrome, and the brown tumor of hyperparathyroidism [2,9-11]. Unlike these lesions, posterior SBC is characterized by its typical location, clear cortical bone margins, asymptomatic nature, and absence of dental involvement. Conversely, anterior SBC, located in the canine to premolar region, is extremely rare. The most recent review (March 2025) [12] reported fewer than 40 cases documented in the English-language literature. Unlike posterior SBCs associated with the submandibular gland, anterior SBCs typically consist mainly of salivary gland tissue (predominantly sublingual gland), with possible inclusion of lymphoid, adipose, vascular, and muscular components, as reported by MRI [6,13] and histopathology [4,14].

SBC occurring from the mandibular incisor region to the premolars differs from typical SBC arising posteriorly in the mandible. When lesions are present near the apical regions of mandibular teeth, panoramic X-rays require further differential diagnosis to distinguish them from tooth-related lesions. Diagnosing SBCs based on panoramic X-rays alone and making an incorrect diagnosis can lead to unnecessary invasive procedures, such as root canal treatment, for conditions that would otherwise only require observation.

Diagnosis primarily relies on CT, MRI, and biopsy. CT findings of SBC are characterized by a single-celled, lingual-opening, submandibular-lingual, round or oval cavity with a cortical rim, where the cavity margins are relatively sharp and regular [5,7,15]. MRI is useful for confirming the continuity between the internal tissue of the SBC and the surrounding salivary glands, potentially avoiding surgical intervention [16].

The etiology of anterior SBC remains unclear. While theories such as hyperactivity of specific muscle groups around the lesion and vitamin D3 deficiency have been proposed [13], the most widely accepted cause is the formation of a cavity due to localized compression of the lingual alveolar bone of the mandible by an adjacent salivary gland [17]. Therefore, careful evaluation using advanced imaging techniques is essential. CT and CBCT provide detailed information on cortical depressions, while MRI confirms continuity between the lesion and adjacent salivary glands. These methods enable definitive diagnosis without invasive or surgical techniques.

Stafne bone cavities, also known as static bone cavities, show no active changes regardless of their location. Furthermore, some recent reports indicate that Type III cases [18,19], which cause bulging and partial fracture of the buccal cortical bone, have been reported, albeit extremely rarely. Therefore, SBCs require careful observation of lesion changes through regular follow-up examinations.

The lesion in this case measured approximately 20.9 × 8.7 × 11.2 mm, relatively large even among previously reported anterior-type SBCs. The CT values also suggested involvement not only of the salivary gland but also of the surrounding soft tissue.

Diagnosing an anterior-type SBC is crucial to avoid unnecessary surgical intervention and patient anxiety. Considering it is not associated with a long-term course and asymptomatic nature, regular X-ray monitoring is considered appropriate disease management. This case adds new insights to the limited literature on anterior-type SBCs and emphasizes the importance of advanced imaging diagnosis in distinguishing this condition from pathological mandibular lesions.

## Conclusions

To prevent misdiagnosis and unnecessary surgical intervention, it is essential to recognize the pathology of anterior-type SBC, as it represents an extremely rare variant of bone defect and may closely resemble odontogenic or non-odontogenic cysts on conventional radiographs. Advanced imaging techniques such as CT, CBCT, and MRI can definitively confirm the diagnosis by demonstrating continuity with adjacent salivary gland tissue and internal structures. This case emphasizes the importance of considering anterior-type SBC in the differential diagnosis of radiolucent lesions in the mandibular anterior and premolar regions, and reaffirms that conservative treatment with clinical and imaging follow-up remains the first-line approach.
